# Exosomal MicroRNA Levels Associated with Immune Checkpoint Inhibitor Therapy in Clear Cell Renal Cell Carcinoma

**DOI:** 10.3390/biomedicines11030801

**Published:** 2023-03-06

**Authors:** Elizaveta Ivanova, Dilara Asadullina, Gulshat Gilyazova, Radmir Rakhimov, Adel Izmailov, Valentin Pavlov, Elza Khusnutdinova, Irina Gilyazova

**Affiliations:** 1 Subdivision of the Ufa Federal Research Centre of the Russian Academy of Sciences, Institute of Biochemistry and Genetics, 450054 Ufa, Russia; 2 Institute of Urology and Clinical Oncology, Bashkir State Medical University, 450008 Ufa, Russia; 3 Republican Clinical Oncological Dispensary, 450054 Ufa, Russia

**Keywords:** renal cell carcinoma, ICI therapy, exosomal miRNAs, immune-related adverse events, PD-1, biomarkers

## Abstract

Immunotherapy with immune checkpoint inhibitors (ICIs) has shown high efficiency in clear cell renal cell carcinoma (ccRCC) treatment. However, the response to therapy among patients varies greatly. Modern studies demonstrate the high potential of exosomal miRNAs as diagnostic and prognostic markers in oncopathology. This study aimed to evaluate exosomal miRNA expression profiles of miRNAs-144, -146a, -149, -126, and -155 in patients with clear cell renal cell carcinoma treated with immune checkpoint inhibitors. The study included 35 patients whose venous blood samples were taken before and after ICI therapy. Expression analysis was performed using real-time quantitative PCR. It was demonstrated that the level of microRNA-146a increased after therapy (median(IQR) 12.92(4.06–18.90)) compared with the level before it (median(IQR) 7.15(1.90–10.50); *p*-value = 0.006). On the contrary, microRNA-126 was reduced after therapy with immune checkpoint inhibitors (median(IQR) 0.85(0.55–1.03) vs. 0.48(0.15–0.68) before and after therapy, respectively; *p*-value = 0.0001). In addition, miRNA-146a expression was shown to be reduced in patients with a higher grade of immune-related adverse events (*p*-value = 0.020). The AUC value for the miRNA-146a and miRNA-126 combination was 0.752 (95% CI 0.585–0.918), with the sensitivity at 64.3% and the specificity at 78.9%. Thus, while it can be assumed that miRNA-146a and miRNA-126 can be used as predictors for ICI therapy effectiveness, additional in-depth studies are required.

## 1. Introduction

Immune checkpoint inhibitors (ICIs) have greatly changed the therapeutic outcomes for patients with clear cell renal cell carcinoma (ccRCC) [1]. However, despite breakthroughs in ccRCC treatment, only up to 40% of patients respond to ICIs, and there are still many unclear questions regarding the mechanism underlying antitumor immunity and the mechanisms of the ICIs’ actions. Renal cell carcinoma (RCC) relates to sensitivity to immune system tumors. This feature allows for the use of immunomodulator drugs, in particular, interferon-α or interleukin-2. Studies showed that tumor cells can “escape” from immunological surveillance by the use of specific mechanisms of preventing the development of autoimmune processes and native tissues damage. Efforts to combat such mechanisms led to the development of novel immunotherapeutic medicines [1]. They work via a process regulated by cellular and molecular factors, and a significant role goes to inhibitory T-cell receptors, the so-called immune checkpoints [2,3] PD-1 (programmed cell death pathway 1) and CTLA-4 (cytotoxic T-lymphocyte associated protein 4) [4,5,6,7]. PD-1 is a membrane protein belonging to the CD28/CTLA-4 receptor family of suppressor T-cells. PD-1 plays a key function in T-cell activity suppression and forestalling autoimmune reactions. PD-1–PD-1 ligand interaction results in cell death of cytotoxic lymphocytes in the tumor microenvironment and enhancement of regulatory T-lymphocytes’ activity, which helps the tumor to “escape” from immunological control [8,9,10]. Blockade of PD-1/PD-L1 enhances antitumor immunity by decreasing the immunosuppressive activity of regulatory T-cells and the reactivation of effectory T-cells; it also stimulates the proliferation of memory B-cells. It is assumed that the effect of PD-L1 expression on a drug’s effectiveness when it acts on PD1/PD-L1 varies depending on the specific drug and disease. Despite the fact that in some patients with high-level expression of programmed cell death ligand 1 (PD-L1) in the tumor, there is a greater effectiveness of immunotherapeutic drugs, it does not allow for predicting the presence or absence of a clinical response to treatment. This may be due to a number of factors. It has been determined that the PD-L1 expression varies during tumor progression due to communication between the tumor and the cells of immune system—the action of antitumor therapy, for example—against a background of the use of tyrosine kinase inhibitors that suppress the activity of EGFR or ALK [4]. PD-L1 expression can be also changed during radiation therapy or chemotherapy—the death of malignant cells leads to a massive release of antigens recognized by the body’s immune system [5]. In addition, tumor heterogeneity should also be taken into account. Increased PD-L1 secretion by tumor cells can be a result of mutations in the *WALK*, *TEN*, and *LKB1* genes. Although no mutations associated with the immunotherapy response have been identified, the effectiveness of immunotherapeutic drugs increases as the number of mutations increases in tumor cells [11].

Biomarkers currently used to predict ICIs’ effectiveness, such as PD-L1 and tumor mutation burden, have restricted prognostic possibilities [12]. The cause of false positive and false negative results in determining the PD-L1 and PD-L2 expression in immunohistochemical studies may be changes in their genes. Nowadays, it is well-known that cancer cells and tumor microenvironment cells secrete extracellular vesicle (EV) compounds, such as exosomes and microvesicles (MVs), identified in the body’s biological fluids, including blood, urine, sperm, and others. Exosomes, a class of small membrane vesicles, support tumor progression by transporting proteins, bioactive substances, mRNAs, lncRNAs, microRNAs (miRNAs), and other agents necessary for the vital functioning of cancer cells. Tumor cells excrete EVs that suppress the immune system response, deactivate T lymphocytes and natural killer cells, and promote regulatory T lymphocyte differentiation and tumor growth [13]. EVs also contain nucleic acids (mRNAs, miRNAs, etc). Following this discovery, miRNAs, a sort of small single-stranded non-coding RNA, have become important players in the modulation of «cancer portrait» and it is known that they are involved in the regulation of innate and specific immune responses by controlling the expression of determining factors of immune control points [14].

From this point of view, circulating EVs containing miRNAs can contribute promising information on tumor cell biology and on interactions between tumors and the immune system.

Recent research proved that exosomes are involved in the development of tumor resistance to medicines and radiation therapy [14,15]. Noted properties of exosomes allow for using them as potential biological markers for liquid, minimally invasive biopsy. Studies of inner-content exosomes may clarify the opportunities for early disease detection of malignant tumors and evaluation of thetherapy’s effectiveness. MiRNAs are of interest in cancer immunotherapy due to the fact that they can directly or indirectly influence the expression of immune checkpoint receptors.

In the present study, we examined immune-related exosomal miRNA expression profiles in clear cell renal cell carcinoma patients treated with nivolumab as potential specific biomarkers for the ICI therapy response.

## 2. Materials and Methods

### 2.1. Study Setting and Population

The study included 35 patients with clear cell renal cell carcinoma treated with nivolumab as immune checkpoint inhibitor therapy. The study included all available patients with ccRCC receiving ICI treatment from 2020 to 2023, who lived in the territory of the Republic of Bashkortostan. We used consecutive sampling in the study, a non-probability sampling technique that seeks to include all accessible subjects as part of the sample [16]. Since the Republican Clinical Oncological Dispensary is the only referral hospital for the Republic of Bashkortostan and its adjoining districts, this approach ensured that the sampling during the last 2 years of enrollment was highly representative of the clinical cases in this locality. In addition, we performed power calculation for our results and observed a high power level equal to 83% for the 0.05 two-sided alpha level significance. The inclusion and exclusion criteria of patients were the same as described in a previous study [17]. The National Cancer Institute’s Common Terminology Criteria for Adverse Events, version 4.0, was used for grading immune-related adverse events [18].

The clinical staff of the Republican Clinical Oncological Dispensary, Departments of Oncology and Urology of the Clinic of Bashkir State Medical University, carried out venous blood sampling from ccRCC patients before and after nivolumab treatment. Informed consent was obtained from each patient for the collection of biological material and molecular genetic studies.

### 2.2. Exosome miRNAs’ Isolation and Quantitative PCR

Exosome miRNAs’ isolation from 1 mL blood plasma, cDNA synthesis, and quantitative real-time PCR were performed as described previously [17] using corresponding miRCURY LNA Kits (Qiagen, Hilden, Germany) and the Rotor-Gene Q real-time PCR product detection system (Qiagen, Hilden, Germany). Reactions included miRNA-16 and hsa-miRNA-1228 as reference genes (endogenous control), and UniSp2, UniSp4, UniSp5, UniSp6, and synthetic cel-miRNA-39 as isolation, reverse transcription, and amplification exogenous controls (Qiagen, Hilden, Germany)). Exosomal miRNA levels’ estimation was performed using the 2^−ΔΔCt^ method based on the fact that the difference in the value of the “threshold cycle” (ΔCt) between the gene of interest and the control gene is proportional to the level of relative expression of the gene of interest.

### 2.3. miRNA Target Prediction and Pathway Analysis

MiRNA target prediction was performed using TarBase [19] and MirTarBase [20]. Pathway analysis of predicted target genes was performed using the STRING [21] and ShinyGO [22] online resources.

### 2.4. Statistical Analysis

The equality of the variances in the distribution of signs was checked using the Mann–Whitney U test for comparing groups on a quantitative basis. A paired Wilcoxon signed-rank test was used for pairwise comparison of studied groups. Results data were presented as a median (IQR). Survival curves of the ccRCC patients were plotted with the Kaplan–Meier method and compared through a log-rank test. The diagnostic capability of the studied miRNAs was evaluated using receiver operating characteristic (ROC) curves and the area under the ROC curve (AUC). Calculations were made using GraphPad Prism 6.07 software (v.6.7, GraphPad Software (Dotmatics), San Diego, CA, USA) and R environment (package *pROC* [23], R Foundation for Statistical Computing, Vienna, Austria). *p*-values less than 0.05 were regarded as the statistically significant level.

## 3. Results

### 3.1. Patient Characteristics

This study included 35 patients with clear cell renal cell carcinoma treated with immune checkpoint inhibitor therapy. Expression levels of exosomal miRNA-144, -126, -146a, -155, and -149 were evaluated before and after ICI therapy. Clinical characteristics are presented in Table 1.

### 3.2. Differential Expression of miRNA

**Figure 1:**
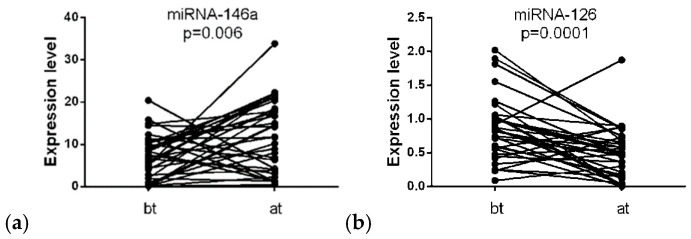

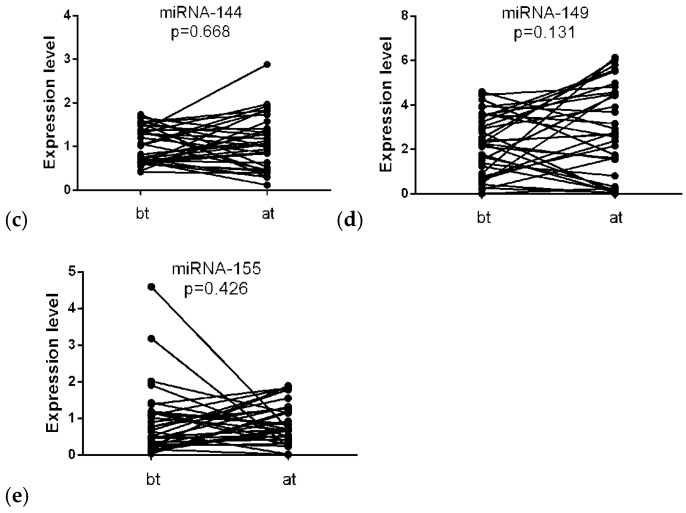
Expression levels of exosomal miRNAs in RCC patients treated with ICI therapy: (**a**) miRNA-146a; (**b**) miRNA-126; (**c**) miRNA-144; (**d**) miRNA-149; (**e**) miRNA-155. Significance level *p*-value was calculated using Wilcoxon test. bt—before therapy; at—after therapy.

We performed expression analysis using quantitative real-time PCR, where dysregulation of exosomal miRNA-126 and miRNA-146a was observed. miRNA-146a levels before ICI therapy were significantly lower compared with levels after therapy (median(IQR): 7.15 (1.90–10.50) and 12.92 (4.06–18.90)), respectively; *p*-value = 0.006) (Figure 1a). In contrast, miRNA-126 expression was higher before therapy compared to expression levels after therapy (median(IQR): 0.85 (0.55–1.03) vs. 0.48 (0.15–0.68), respectively; *p*-value = 0.0001) (Figure 1b). miRNA-149, miRNA-144, and miRNA-155 provided no significant differences in the comparison groups (*p*-value > 0.05) (Figure 1c–e).

### 3.3. Association of miRNA-146a and miRNA-126 Expression Levels with Immune-Related Adverse Events

**Figure 2:**
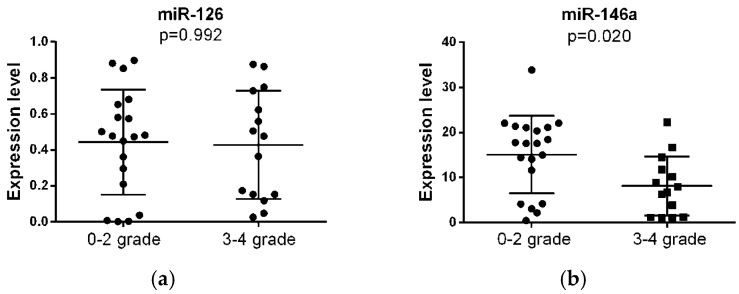
Expression levels of exosomal miRNAs in clear cell renal cell carcinoma patients with different grades of immune-related adverse events after second-line nivolumab therapy: (**a**) miRNA-126; (**b**) miRNA-146a. Significance level *p*-value was calculated using Mann–Whitney U test. 0–2 grade, 3–4 grade—grades of irAEs.

We examined expression levels of miRNA-146a and miRNA-126 concerning immune-related adverse events (irAEs) from nivolumab treatment. There were 20 patients with 0–2 grades of irAEs and 15 with 3–4 grades (Table 2). It was shown that ccRCC patients with 3–4 grades of irAEs had lower miRNA-146a expression (median(IQR): 17.54(6.03–21.07) vs. 7.35(1.21–12.44) for 0–2 grades and 3–4 grades, respectively; *p*-value = 0.02) (Figure 2a). Additionally, miRNA-126 did not show a statistically significant alteration in expression level between groups of patients with high and low irAE grades (Figure 2b).

### 3.4. Association between miRNA-146a Expression and ccRCC Patient Overall Survival

**Figure 3:**
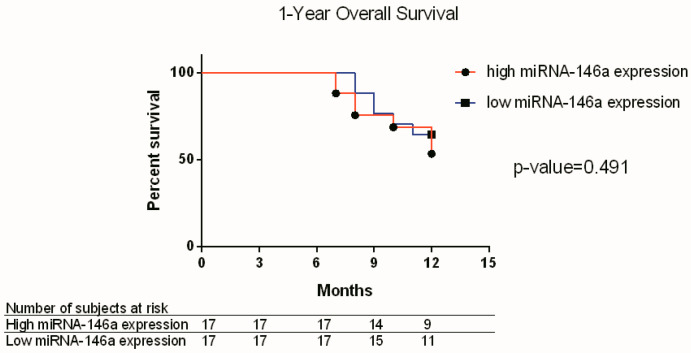
The Kaplan–Meier analysis of high and low miRNA-146a expression level groups of ccRCC patients after ICI therapy. *p*-value was calculated using Gehan–Breslow–Wilcoxon test.

High and low miRNA-146a expression level groups were determined according to the median of miRNA-146a expression in groups of patients after ICI therapy. The rate of 1-year OS of ccRCC patients included in the study was 53.5% in that group with high miRNA-146a expression, and 64.7% in the low miRNA-146a expression group. The Kaplan–Meier analysis did not show a statistically-significant difference (*p*-value = 0.491) in survival (Figure 3) between the comparison groups.

### 3.5. Logistic Regression and ROC Curve Analyses

**Figure 4:**
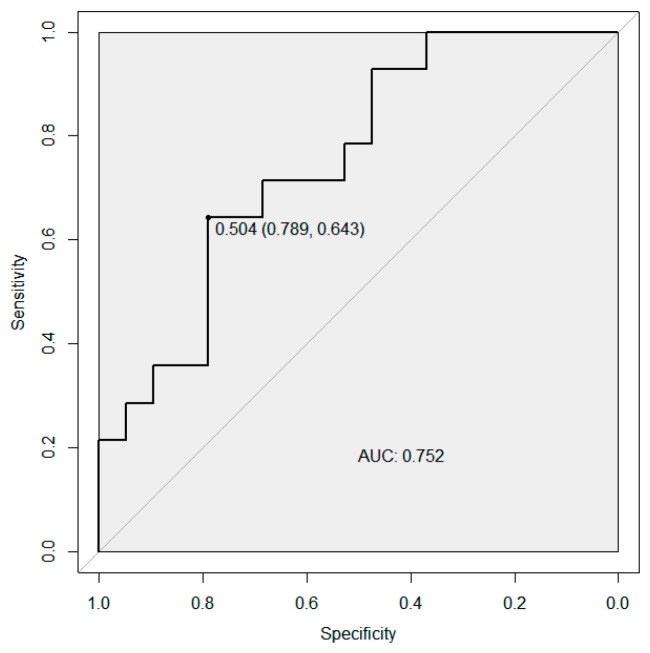
The ROC curve regarding the expression level of the miRNA-146a and miRNA-126 combination to discriminate irAEs grades after ICI therapy in ccRCC patients. ROC—receiver operating characteristic; AUC—area under the curve.

Logistic regression with following ROC curve analyses was performed to evaluate the capability of the miRNA-146a and miRNA-126 combination to discriminate between irAEs grades. As shown in Figure 4, the AUC value for the studied miRNA combination was 0.752 (95% CI 0.585–0.918), with the sensitivity at 64.3% and the specificity at 78.9%.

### 3.6. Pathway Enrichment Analysis

**Figure 5:**
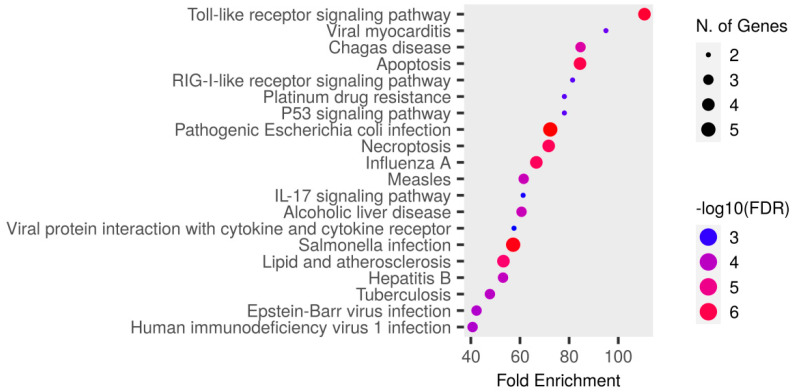
Dot plot showing the results of KEGG pathway enrichment analyses performed for miRNA-146a and miRNA-126 validated targets. N. of Genes- number of genes.

We analyzed validated targets of miRNA-146a and miRNA-126 from the TarBase and MirTarBase databases to better understand the role of these miRNAs in ICI-resistant formations and renal cell carcinoma development. The list of included genes is presented in Table 3. Pathways enrichment analysis of the Kyoto Encyclopedia of Genes and Genomes (KEGG) database identified 15 significantly enriched pathways (Table 4). Among the most significant enriched pathways were the Toll-like receptor signaling pathway, pathogenic Escherichia coli infection, salmonella infection, and apoptosis (Figure 5).

**Figure 6:**
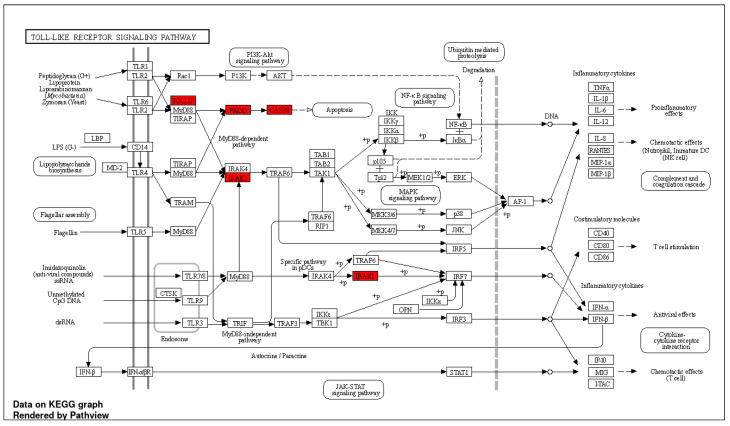
Toll-like receptor signaling pathway including miRNA-146a and miRNA-126 validated target genes (highlighted in red), according to the KEGG database.

The most significant enriched pathway was the Toll-like receptor signaling pathway, including four of the analyzed genes: *TOLLIP*, *FADD*, *CASP8*, and *IRAK1* (Figure 6).

**Figure 7:**
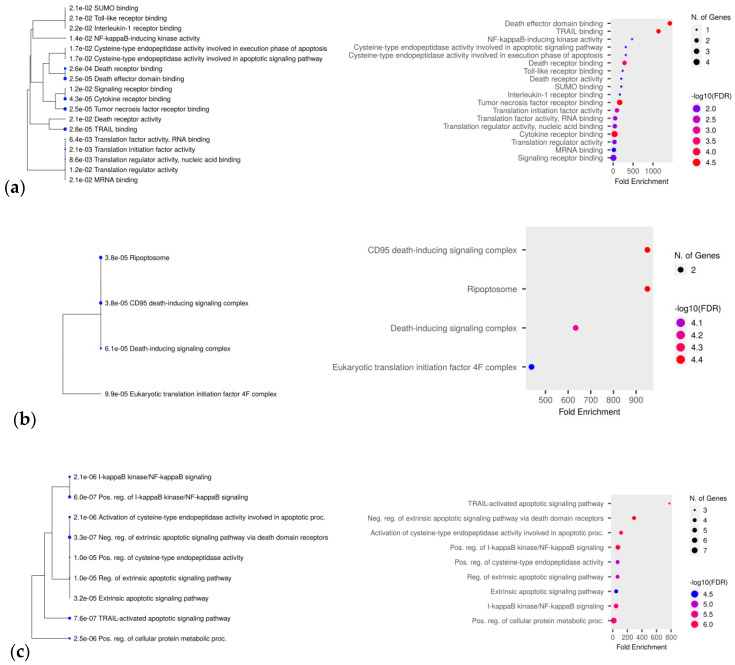
Gene Ontology (GO) function pathways of gene set through ShinyGO. (**a**) Tree and dot plots of GO molecular function; (**b**) cell component; (**c**) biological processes.

Gene Ontology (GO) analysis was conducted to assess the involvement of the miRNA-146a and miRNA-126 validated target genes in the relevant biological process (BP), cellular component (CC), and molecular function (MF)—which are all summarized in Figure 7. Molecular function pathways were enriched by the next most significant pathways: death effector domain binding, TRAIL binding, NF-kappaB-inducing kinase activity, tumor necrosis factor receptor binding, and cytokine receptor binding (Figure 7a). Among cell components, the most significant pathways included CD95 death-inducing signaling complex, ripoptosome, and eukaryotic translation initiation factor 4F complex (Figure 7b). Concerning BP, the identified genes were significantly involved in the TRAIL-activated apoptotic signaling pathway, negative regulation of extrinsic apoptotic signaling pathway via death domain receptors, and positive regulation of I-kappaB kinase/NF-kappaB signaling, among others (Figure 7c).

## 4. Discussion

Immune checkpoint inhibitors are often used in modern clinical practice. However, optimal selection of patients to achieve the effect of immunotherapy using non-invasive biomarkers is difficult. ICIs have expanded the possibilities of cancer therapy, and nowadays, they are applied in approximately 50 different cancer types [24]. Therefore, increasing attention has been paid to the identification and development of predictive biomarkers for the response of ICIs. Such biomarkers will allow to determine responders and non-responders to ICIs before the therapy initiation. In this way, miRNAs are attractive as high-potential prognostic, diagnostic biomarkers in different cancer types, response treatments, and drug resistance scenarios. miRNA expression profiles may be applied to evaluate the molecular-based findings resulting from ICI therapy and forecast the therapy response. miRNAs are known to have a role in intercellular communication in cancer. We supposed that exosomal miRNAs are potential molecular agents that allow ICI drugs to exhibit their antitumor activity.

Exosomal microRNA expression profile analysis during ICI therapy in patients with ccRCC has not been previously studied. There are studies demonstrating aberrant expression of circulating miRNAs during ICI therapy in lung cancer and melanoma patients [25,26,27,28,29]. Halvorsen and colleagues determined 7 microRNAs in serum connected with OS in lung cancer patients treated with nivolumab [25]. Using expression levels of exosomal miRNAs as a criterion for patient selection before checkpoint inhibitor therapy in NSCLC was studied by Peng. miRNAs belonging to the hsa-miR-320 family were upregulated and associated with an unfavorable response to PD-1 inhibitors. In contrast, miR-125b-5p expression was reduced in patients with responses to anti-PD-1 therapy. The authors considered the use of mir-125 expression as an on-therapy diagnostic tool for response monitoring [26]. Another study also demonstrated an association between decreased expression of microRNA-320 and microRNA-375 and the clinical benefit of nivolumab therapy in advanced non-small-cell lung cancer patients. The clinical effectiveness was attested with increases in exoPD-L1 and the PD1 + CD8 + T-cell fraction and a decrease in immunosuppressive cytokines, which indicated the connection of miRNA expression with these processes [28]. Francesco Pantano and colleagues demonstrated that the pretreatment level of extracellular vesicle-associated miR-625-5p was related to survival values in ICI-treated NSCLC patients. In spite of the correlation of the miR-625-5p expression level with high PD-L1 expression, this made it possible to identify ICI non-responders, despite their PDL-1 expression of more than 50% [27]. Li et al. investigated exosomal miR-3913-5p and miR-184 expression levels significantly upregulated after the onset of osimertinib non-response in NSCLC patients [29]. The eight specific miRNAs signatures were highly-increased by nivolumab therapy and were attenuated only in peripheral lymphocytes for long-responder metastatic ccRCC patients [30].

MiRNA-146a plays the regulatory role in perforin and IFN-γ development in T-cells, thus reducing severe immune-related adverse events (irAEs) [31]. Previously, we demonstrated that reduced expression of miRNA-146 in patients with ccRCC with serious immune-related adverse events and SNP rs2910164 correlated with a higher risk of severe irAE production [17]. It is also known that glatiramer acetate, used for RRMS treatment, promotes the restoration of the aberrant expression of miR-142-3p and miR-146a in mononuclear cells of peripheral blood from untreated RRMS patients [32].

miRNA-146a participates in the regulation of the pro-inflammatory immune response and is controlled by the NF-kB gene. miR-146a is aberrantly expressed in various diseases and regulates processes of the inflammatory response, innate and adaptive immunity, differentiation of monocytic lines, and tumorigenesis [33]. It was previously determined that the miRNA signature, including miRNA-146a, is involved in the accumulation of myeloid-derived suppressor cells (MDSCs) and the development of treatment resistance to immune checkpoint inhibitor therapy in patients with melanoma [34]. MDSCs directly organize the mobility of cancer cells, causing the EMT [35] and stimulating signaling pathways related to resistance to therapy, angiogenesis, and stroma restructuring [36].

We have not found any literature on the analysis of the miR-126 expression profile in the ICI therapy. miRNA-126 deregulation has been detected in different cancer types. It is known that miRNA-126 controls angiogenesis by regulating genes involved in phosphatidylinositol 3-kinase pathways, the vascular endothelial growth and carcinogenesis pathways where miRNA-126 plays a dual role as oncogene or suppressor [37,38,39,40,41,42,43,44,45,46]. miRNA-126 downregulation correlated with a shorter time until disease recurrence in ccRCC patients [40]. Additionally, decreased miRNA-126 expression was observed in metastatic tumors in comparison with primary ccRCC tumors [38,40,47,48]. Agudo and colleagues identified that miRNA-126 regulates innate immunity—managing the expression of vascular endothelial growth factor receptor 2 (VEGFR2) expressed by pDCs and the mTOR signaling pathway—which controls VEGFR2 expression through the miRNA-126–TSC1 interplay [45,49]. Plasmacytoid dendritic cells constitute mainstream components of the immune response and perform an important role in the modulation of immune tolerance [43]. Downregulated miR-126 could promote carcinogenesis due to the decreased amount of IFN-α produced by pDCs [50]. At another point, miRNA-126 upregulation may increase chronic inflammation and autoimmunity [51]. Lehman et al. determined that stem cell spreading is regulated by miRNA-126 via the PI3K/AKT/GSK3 signaling pathway. miR-126 overexpression disrupts cell cycle start, leading to a decrease in hematopoietic contribution. miRNA-126 controls a lot of targets within the PI3K/AKT/GSK3β pathway, reducing signal transmission in response to external signals [52]. miRNA-126 expression in CD4 + T-cells was increased in relapsing–remitting multiple sclerosis (RRMS) patients and downregulated on treatment with natalizumab [53]. Natalizumab-mediated miRNA-126 regulation likely supports a speedy and stable slowdown in disease activity.

This may explain our obtained results, which indicate that exosomal miRNA-126-5p expression is an essential controller of inflammatory reactions and immune response. The limited samples, interpopulation differences, different sources of miRNA, tumor heterogeneity, and various localizations of tumors may explain the ranging results of the above studies. The current study also had a small group of participants as a limitation factor, but still had sufficient power. Further associated studies in large patient groups are needed to search for a biomarker to forecast the response to ICI therapy in ccRCC patients.

## 5. Conclusions

Our results showed differences in the exosomal miRNA-146a and miRNA-126 expression levels involved in the immune response regulation in ccRCC patients before and after ICI therapy. Understanding the mechanisms underlying the individual characteristics of the ICI therapy response remains one of the key tasks to developing a personalized approach in cancer therapy. The data obtained from small samples confirm the role of exosomal miRNAs in predicting the ICI therapy’s effectiveness. Further studies and data validation on large samples will allow for the further use of exosomal miRNAs expression profiles as biomarkers for predicting the effectiveness and safety of immunotherapy.

## Figures and Tables

**Table 1:** Baseline patient characteristics.

Characteristics	№ of Patients	%
Age, years	62	
Median	41–79	
Gender		
Male	16	45.7
Female	19	54.3
Histology		
Clear cell	35	100.0
Non-clear cell	0	0
IMDC risk classification		
Favorable	2	5.71
Intermediate	25	71.43
Poor	8	22.86

IMDC: International Metastatic Renal Cell Carcinoma Database Consortium.

**Table 2:** IrAEs in RCC patients according to category and grade.

Category	Grading, Number of Patients	
	Grades 0–2, n (%)	Grades 3–4, n (%)
Skin-related events	7 (35.0)	5 (33.3)
Pneumonitis	1 (5.0)	5 (33.3)
Diarrhea/colitis	5 (25.0)	2 (13.3)
Endocrine-related events	12 (60.0)	3 (20.0)
Pancreatitis	1 (5.0)	2 (13.3)
Colitis	2 (10.0)	0
Hepatitis	1 (5.0)	1 (6.67)
Nephritis	1 (5.0)	0
Myalgia	1 (5.0)	0
Joint pain	1 (5.0)	0
Others	0	2 (13.3)

**Table 3:** Validated miRNA-146a and miRNA-126 target genes included in enrichment analysis.

Symbol	Ensembl Gene ID	Chr	Position (Mbp)	Description
*CASP8*	ENSG00000064012	2	201.2334	caspase 8
*TNFSF10*	ENSG00000121858	3	172.5055	TNF superfamily member 10
*TNFRSF10B*	ENSG00000120889	8	23.0201	TNF receptor superfamily member 10b
*TOLLIP*	ENSG00000078902	11	1.2744	Toll-interacting protein
*EIF4G2*	ENSG00000110321	11	10.7971	eukaryotic translation initiation factor 4 gamma 2
*FADD*	ENSG00000168040	11	70.2033	Fas-associated death domain
*EIF4A1*	ENSG00000161960	17	7.5728	eukaryotic translation initiation factor 4A1
*IRAK1*	ENSG00000184216	X	154.0105	Interleukin-1 receptor-associated kinase 1

**Table 4:** Enriched KEGG pathways for miRNA-146a and miRNA-126 validated targets.

Ontology	Description	Strength	False Discovery Rate
hsa04620	Toll-like receptor signaling pathway	1.57	0.00052
hsa05130	Pathogenic Escherichia coli infection	1.4	0.00052
hsa05132	Salmonella infection	1.35	0.00052
hsa04210	Apoptosis	1.45	0.0010
hsa04217	Necroptosis	1.4	0.0013
hsa05164	Influenza A	1.35	0.0016
hsa05142	Chagas disease	1.45	0.0082
hsa04668	TNF signaling pathway	1.4	0.0102
hsa05162	Measles	1.31	0.0165
hsa04215	Apoptosis—multiple species	1.79	0.0179
hsa05161	Hepatitis B	1.25	0.0202
hsa05152	Tuberculosis	1.22	0.0217
hsa05169	Epstein–Barr virus infection	1.16	0.0297
hsa05170	Human immunodeficiency virus 1 infection	1.14	0.0323
hsa05416	Viral myocarditis	1.53	0.0378

## Data Availability

The data used and obtained during the study are available from the corresponding author on request.
